# Impact of Pretreatment Systemic Inflammatory Markers on Treatment Persistence with Biologics and Conventional Systemic Therapy: A Retrospective Study of Patients with Psoriasis Vulgaris and Psoriatic Arthritis

**DOI:** 10.3390/jcm12083046

**Published:** 2023-04-21

**Authors:** Eiki Sugimoto, Hiroki Matsuda, Sayaka Shibata, Yuka Mizuno, Asumi Koyama, Lixin Li, Haruka Taira, Yukiko Ito, Kentaro Awaji, Takashi Yamashita, Shinichi Sato

**Affiliations:** Department of Dermatology, Graduate School of Medicine, The University of Tokyo, Tokyo 113-8655, Japan

**Keywords:** biologics, conventional systemic agents, PLR, SII, treatment response

## Abstract

Systemic inflammation plays a central role in the pathophysiology of psoriasis. This study examined accessible systemic inflammatory markers in patients with psoriasis vulgaris and psoriatic arthritis. We aimed to evaluate their association with psoriasis severity, the presence of arthritis, and drug continuation rates. The findings revealed that neutrophil, monocyte, and platelet count, neutrophil/lymphocyte ratio, monocyte/lymphocyte ratio, *systemic* inflammation response index, *systemic* immune/inflammation index (SII), and CRP were positively correlated with *Psoriasis* Area and Severity Index scores. Patients presenting with higher platelet/lymphocyte ratio (PLR) or CRP values were more likely to be diagnosed with psoriatic arthritis than with psoriasis vulgaris in the multivariate regression analysis. Importantly, patients with higher pretreatment neutrophil or platelet count, PLR, and SII were associated with lower treatment continuation rates of conventional systemic agents. Higher pretreatment scores of systemic inflammatory markers did not affect treatment retention rates of biologics. These findings suggest that several accessible systemic inflammatory markers may effectively assess underlying systemic inflammation and may provide an indication for a therapeutic approach in patients with psoriasis vulgaris and psoriatic arthritis.

## 1. Introduction

Psoriasis is a common chronic inflammatory skin disease characterized by well-demarcated scaly thick erythematous plaques, leading to a reduced quality of life [1,2]. Psoriasis is associated with systemic inflammation and has been linked to various comorbidities, including arthritis, metabolic syndrome, cardiovascular disease, and depression. Systemic agents, both biologics and conventional systemic agents, have favorable treatment outcomes with a notable reduction in systemic inflammation [3,4,5].

The disease concept of “psoriatic disease (PsD)” has been established, signifying that inflammation in psoriasis extends beyond the skin to affect a wide variety of organs, including joints, blood vessels, heart, and brain [6]. Various pathological conditions such as dermatitis, arthritis, and metabolic syndrome may be affected by the underlying systemic inflammation. The systemic inflammation in psoriasis is fueled partially by inflammatory cytokines and adipokines produced by visceral adipose tissue [7,8,9]. Dysregulation of adipokines, including adiponectin, leads to dysfunction in vascular endothelial cells and predisposes to the formation of atherosclerotic plaques, increasing the risk of cardiovascular events and finally leading to the exacerbation of a series of inflammatory processes known as the psoriatic march [10,11]. In addition to systemic inflammation resulting from adipocyte or vascular dysfunction, a common infiltration of Th17 cells and a similar cytokine profile with elevated Th17-related factors have been detected in skin, joints, and atherosclerotic vascular lesions [12,13,14]. This suggests that systemic inflammation may be multiorgan in nature and also from an immunological perspective.

Clinical evidence is accumulating that increased and sustained systemic inflammatory status of psoriasis patients is a critical determinant that can affect the disease outcome of PsD [15,16]. Several inflammatory- and immune-based scores have been developed to monitor the status of systemic inflammatory status [17]. These include neutrophil-to-lymphocyte ratio (NLR), monocyte-to-lymphocyte ratio (MLR), and platelet-to-lymphocyte ratio (PLR), which have been reported to be elevated in psoriasis patients and to be correlated with Psoriasis Area and Severity Index (PASI) scores [18,19]. More recently, systemic immuno-inflammatory index (SII) and systemic inflammatory response index (SIRI) are also known as scores that may reflect systemic inflammation more comprehensively. These new scores have been established as effective predictors of prognosis in neoplastic and cardiovascular diseases; however, their significance in psoriasis is still poorly evaluated [20,21]. These inflammatory markers are readily available and can be quantitatively assessed, and thus, it would be significant if they reflect disease activity and predict treatment responsiveness.

The present study evaluated peripheral blood parameters and systemic inflammatory scores in psoriasis patients with or without arthritis compared with healthy controls. We examined the association of each systemic score system with PASI scores and with the presence of arthritis. We further explored the potential of systemic inflammatory scores as a predictor of treatment response in psoriasis by analyzing the association between these scores and continuation rates of systemic therapy.

## 2. Methods

### 2.1. Patients

A retrospective analysis was performed on psoriasis patients who first visited the University of Tokyo Hospital (Tokyo, Japan) from April 2019 to March 2022. All patients enrolled in this study were given diagnoses of psoriasis vulgaris (PsV) or psoriatic arthritis (PsA) by dermatologists and rheumatologists according to the Classification Criteria for Psoriatic Arthritis (CASPAR) criteria. Patients with generalized pustular psoriasis, guttate psoriasis, and palmoplantar pustulosis were not included in the present study. Healthy controls had no history of allergy or skin diseases, including atopic dermatitis. The medical ethics committee of the University of Tokyo approved all described studies (No. 3360), and the study was conducted according to the principles of the Declaration of Helsinki.

Patient treatment included topical, oral, ultraviolet therapies, and biologics. Oral therapy included etretinate, apremilast, cyclosporine, and methotrexate. Biologics included inhibitors for TNF-a, IL-17A, IL-17 receptor, and IL-23p19. TNF inhibitors included infliximab and adalimumab; IL-17 inhibitors included the IL-17A inhibitors secukinumab and ixekizumab and the IL-17 receptor inhibitor brodalumab; and IL-23 inhibitors included the IL-23p19 inhibitors guselkumab, tildrakizumab, and risankizumab. Patients treated with the JAK inhibitor upadacitinib, the TNF inhibitor certolizumab pegol, the IL-17A/IL-17F inhibitor bimekizumab, and the IL-12/23 inhibitor ustekinumab were not included in the present study. All patients were treated with the prescribed protocol based on insurance coverage. The patients were excluded if they showed any symptoms of infection at the time of diagnosis and data collection.

### 2.2. Clinical Assessments and Data Collection

The hematological laboratory data of patients and healthy controls were extracted from our registry created at the time of diagnosis. The severity of the skin was evaluated by PASI scores [22]. PsA patients were classified as having peripheral or axial arthritis or a combination of both diseases.

### 2.3. Definition of Systemic Inflammatory Markers

Each systemic inflammatory score was calculated as follows:

NLR = absolute neutrophil count (×10^9^/L)/absolute lymphocyte count (×10^9^/L)

MLR = absolute monocyte count (×10^9^/L)/absolute lymphocyte count (×10^9^/L)

PLR = absolute platelet count (×10^9^/L)/absolute lymphocyte count (×10^9^/L)

SII = absolute neutrophil count (×10^9^/L) × absolute platelet count (×10^9^/L)/absolute lymphocyte count (×10^9^/L)

SIRI = absolute neutrophil count (×10^9^/L) × absolute monocyte count (×10^9^/L)/absolute lymphocyte count (×10^9^/L).

### 2.4. Statistical Analysis

Statistical analysis was performed by Kruskal–Wallis test with Dunn–Bonferroni post hoc test for multiple comparisons for the items regarding age, cell counts for neutrophils, lymphocytes, monocytes, and platelets and NLR, MLR, PLR, SII, and SIRI. Mann–Whitney’s U-test was used for two-group comparisons. Fisher’s exact test for frequency comparison was used for group comparisons. Spearman’s rank correlation test was used to examine the relationship between two continuous variables. Spearman’s correlation method was conducted to determine correlation coefficients for ten inflammatory markers. Regarding the cut-off values for systemic inflammatory markers, the area under the curve (AUC) was calculated and optimal cut-off values were determined using the Youden Index from the receiver operating characteristic (ROC) curve [23]. Univariate and multivariate Cox regression analyses were conducted to analyze the association between inflammatory markers and diagnosis of PsA. All variables were included in both univariate and multivariate regression model. The Kaplan–Meier method and log-rank test were used to compare the two groups’ continuation rates of psoriasis treatment. Cut-off values of systemic inflammatory markers for the treatment persistence were set to the mean + 2SD of healthy controls. *p* < 0.05 was considered statistically significant throughout all the analyses. The statistical data were generated using the Prism 9 software program (Graph Pad Software, San Diego, CA, USA) and the JMP^®^ Pro 17.0.0 (SAS Institute Inc., Cary, NC, USA).

## 3. Results

### 3.1. Baseline Characteristics and Systemic Inflammatory Markers

A total of 164 patients (117 patients with psoriasis vulgaris (the PsV group) and 47 patients with psoriatic arthritis (the PsA group)) and 50 healthy controls (the Healthy group) were enrolled in the present study. The sex and mean age of patients with psoriasis vulgaris, those with psoriatic arthritis, and healthy controls were as follows: 80 men and 37 women for patients with psoriasis vulgaris with a mean age of 51.16 ± 18.26 years, 29 men and 18 women for patients with psoriatic arthritis with a mean age of 55.49 ± 13.78 years, and 32 men and 18 women for healthy controls a mean age of 54.30 ± 13.62 years. The sex and age of the PsV, the PsA and the Healthy group were not significantly different between the groups (Table 1). Although no significant difference was observed between the PsV and the Healthy group for six peripheral blood parameters (neutrophil counts, lymphocyte counts, monocyte counts, platelet counts) and for all of the five calculated systemic inflammatory markers (NLR, MLR, PLR, SII, and SIRI) and CRP. Monocyte counts were significantly higher in the PsA group (0.437 ± 0.154) compared with the Healthy group (0.368 ± 0.111, *p* = 0.0179). Compared to the PsV group, the PsA group showed a significant increase in platelet counts (259.52 ± 71.14 for PsV group vs.289.85 ± 90.13 for PsA group, *p* = 0.0324) and C-reactive protein (CRP) (0.29 ± 0.57 for PsV group vs.1.48 ± 4.84 for PsA group, *p* = 0.0284) (Table 1). All the other items examined were not significantly different between the groups.

### 3.2. Correlations of Systemic Inflammatory Biomarkers with PASI Scores

**Figure 1:**
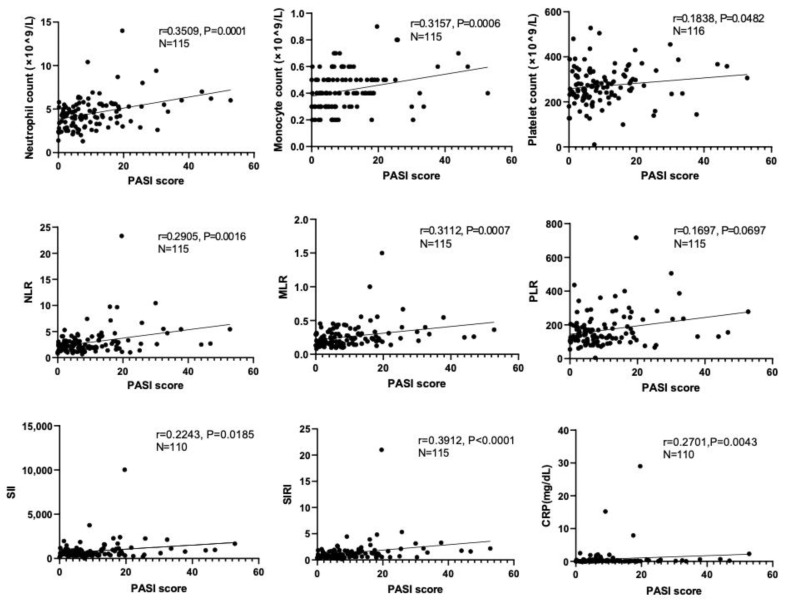
Correlations between peripheral blood parameters or systemic inflammatory markers and PASI scores are shown. Solid lines indicate linear regression lines. Spearman’s rank correlation coefficient (r) was used for correlation analyses.

**Figure 2:**
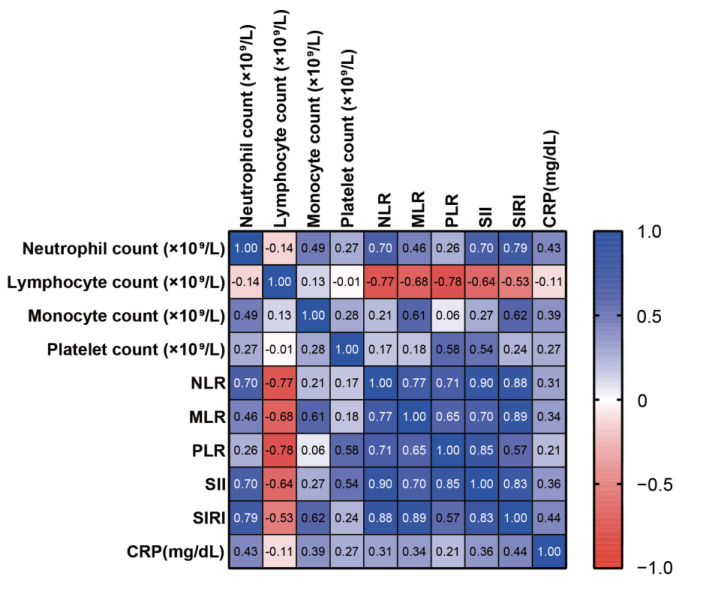
Spearman’s correlation coefficients among four peripheral blood parameters and six systemic inflammatory markers. Blue boxes indicate positive correlations, and red boxes indicate negative correlations.

We next examined whether peripheral blood parameters and systemic inflammatory markers had any correlation with disease severity. PASI scores from both the PsV and PsA group were analyzed for the association. As shown in Figure 1, neutrophil count (*p* = 0.0001, r = 0.3509), monocyte count (*p* = 0.0006, r = 0.3157), platelet count (*p* = 0.0482, r = 0.1838), NLR (*p* = 0.0016, r = 0.2905), MLR (*p* = 0.0007, r = 0.3112), SII (*p* = 0.0185, r = 0.2243), SIRI (*p <* 0.0001, r = 0.3912), and CRP (*p* = 0.0043, r = 0.2701) were positively correlated with PASI scores. We also performed Spearman’s correlation method to determine correlation coefficients for ten inflammatory markers (four peripheral blood parameters and six systemic inflammatory markers). High positive correlations were found among these inflammatory markers, especially between SII and PLR (r = 0.85), SII and NLR (r = 0.90), SIRI and MLR (r = 0.89), SIRI and NLR (r = 0.88), and SII and SIRI (r = 0.83) (Figure 2). Negative correlations were found between lymphocyte count and neutrophil count (r = −0.14), NLR (r = −0.77), MLR (r = −0.68), PLR (r = −0.78), SII (r = −0.64), SIRI (r = −0.53), and CRP (r = −0.11) (Figure 2).

### 3.3. Association between Systemic Inflammatory Markers and Diagnosis of PsA

We next examined whether six systemic inflammatory markers at initial presentation could predict the presence of arthritis. The area under the curve (AUC) was determined, and optimal cut-off values were calculated using the Youden Index from the receiver operating characteristic (ROC) curve [23] (Table 2). A Cox regression proportional hazard analysis was performed to compare the diagnosis of psoriatic arthritis versus psoriasis vulgaris based on NLR, MLR, PLR, SIRI, SII, and CRP values. All variables were included in both univariate and multivariate regression model. The results showed that MLR (OR = 2.355, *p* = 0.039), PLR (OR = 5.775, *p* = 0.005), SIRI (OR = 2.423, *p* = 0.044) and CRP (OR = 3.251, *p* = 0.008) were associated with a higher probability of diagnosis with psoriatic arthritis by univariate analysis (Table 3). The analysis revealed that the association between PLR and CRP with the diagnosis of psoriatic arthritis remained statistically significant in the multivariate analysis (PLR: OR = 7.027, *p* = 0.040; CRP: OR = 3.179 *p* = 0.022; Table 3). Thus, patients with higher PLR or CRP values at the time of initial presentation were more likely to be diagnosed with psoriatic arthritis than with psoriasis vulgaris.

### 3.4. Characteristics and Systemic Inflammatory Markers among Patients with Psoriatic Arthritis with or without Axial Lesions

Patients with psoriatic arthritis exhibit joint inflammation in both their peripheral and axial lesions. We investigated whether there were any differences in patients’ characteristics, peripheral blood counts, or systemic inflammatory markers, including erythrocyte sedimentation rate (ESR), between patients with and without axial lesions (Table 4). Results showed that patients with axial lesions had higher PASI scores (12.02 ± 9.75) compared to those without (6.05 ± 5.52). In addition, CRP (2.60 ± 6.55 for patients with axial lesions vs. 0.26 ± 0.53 for patients without axial lesions, *p* = 0.0271) were statistically higher in patients with axial lesions compared to those without. No significant differences were found in baseline characteristics, peripheral blood counts and other systemic inflammatory markers between the two groups. A Cox regression proportional hazard analysis was conducted to compare the relationship between the presence of axial lesions and systemic inflammatory markers, including NLR, MLR, PLR, SIRI, SII, and CRP; however, no significant association was detected.

### 3.5. Association between Systemic Inflammatory Markers and Treatment Continuation Rates of Biologics and Conventional Systemic Agents

We next evaluated whether pretreatment peripheral blood parameters or systemic inflammatory markers were associated with treatment continuation rates. Since patients who initiated topical therapy were not followed in all cases, subsequent analyses examined treatment persistence for patients who initiated systemic therapy. A list of each systemic therapy and the number of patients is provided in Table 5. No patients used the biologics of certolizumab pegol, bimekizumab or ustekinumab, or JAK inhibitors as initial therapy. All patients were started on monotherapy with either biologics or conventional systemic agents. The study included 51 patients in the biologics group and 48 patients in the conventional systemic therapy group. The treatment continuation rate of these patients during the first year of treatment was evaluated. During the one-year follow-up, several patients received additional concomitant drugs, which are listed in the right row of Table 5. Patients treated with biologics or conventional systemic agents included both PsV and PsA patients (60 PsV and 39 PsA patients).

Cut-off values of peripheral blood parameters or systemic inflammatory markers were set to the mean + 2SD of healthy controls (Table 6). Patients were divided into two groups by pretreatment scores according to the cut-off values. The median treatment durations were compared between the two groups by log-rank tests.

First, we examined whether there was a difference in the treatment retention rate of biologics between the two groups divided by the cut-off value of pretreatment scores. As shown in Table 6, patients treated with biologics generally exhibited high treatment persistence, with a median treatment duration of more than 300 days. Treatment persistence was comparable for all systemic inflammatory markers, regardless of pretreatment high or non-high scores.

**Figure 3:**
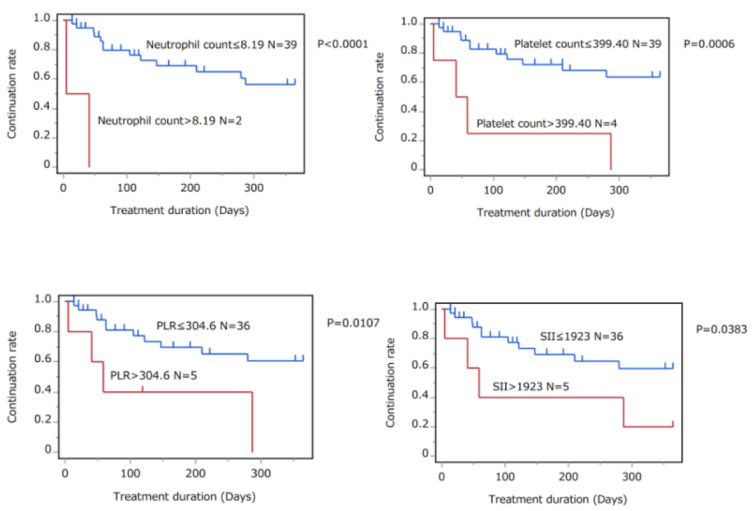
Kaplan–Meier curves for treatment continuation rates of patients who received treatment with oral apremilast, methotrexate, cyclosporine, and etretinate. Patients were divided into two groups by pretreatment scores according to the cut-off values and determined by mean + 2SD levels of healthy controls (neutrophil counts; >8.19 × 10^9^/L, N = 2 and ≤8.19 × 10^9^/L, N = 39, platelet counts; >399.40 × 10^9^/L, N = 4 and ≤399.40, N = 39, PLR; >304.6, N = 5 and ≤304.6, N = 36, and SII; >1923, N = 5 and ≤1923, N = 36). The continuation rates of the two groups were compared by log-rank test.

Next, the study focused on the patients who initiated treatment with oral apremilast, methotrexate, cyclosporine and etretinate. These patients were then evaluated for their treatment continuation rates between the two groups divided by the cut-off value of pretreatment scores. The results of Kaplan–Meier analyses by log-rank tests showed that patients above the cut-off values for neutrophil counts, platelet counts, PLR, and SII exhibited significantly lower treatment continuation rates (Figure 3 and Table 7). Other parameters were also examined; however, no significant differences were detected between the high-score and non-high-score groups (Table 7).

## 4. Discussion

The present study examined new inflammatory markers such as SII and SIRI in Japanese patients with PsV and PsA. SII and SIRI tended to be higher in patients with PsA compared with those with PsV, although the differences were not significant in the present study. Importantly, patients with higher levels of PLR or CRP at initial presentation were more likely to be diagnosed with PsA, suggesting that these markers may be a diagnostic help for the presence of arthritis. In addition, we examined whether higher scores of systemic inflammatory markers may affect continuation rates of systemic treatment of biologics and conventional systemic agents. The current study, for the first time, revealed that patients with higher platelet or neutrophil counts, PLR, and SII scores exhibited lower treatment continuation rates for conventional systemic agents. This was not the case with patients treated with biologics, and patients with biologics generally showed high treatment persistence regardless of pretreatment systemic inflammatory scores in this study.

Psoriasis is an immune-mediated inflammatory disease with underlying systemic inflammation which affects various organs beyond the skin. Although the visible manifestation of systemic inflammation is dermatitis, other associated conditions, including arthritis, cardiovascular diseases, metabolic syndrome, and psychiatric disorders, have been linked to systemic inflammation [6,24]. Several reports have demonstrated that patients with these inflammatory conditions, even those without psoriasis, exhibit elevated systemic inflammatory scores [25,26,27,28,29]. Therefore, the excessive inflammation with comorbid conditions across multiple organs in psoriasis patients may further aggravate systemic inflammation [18,30]. Hidradenitis suppurativa (HS) is a chronic inflammatory disease that causes painful and swollen nodules. Similar to psoriasis, HS patients exhibit combordities such as obesity and metabolic syndrome, with impaired adipokine release in its pathogenesis [31,32]. SII and pan-immune-inflammation value (PIV), a more comprehensive inflammatory marker, are elevated in HS patients compared to healthy individuals, and a correlation between systemic inflammatory scores and HS severity has been reported [33]. Thus, patients with psoriasis and HS who have elevated inflammatory markers at initial diagnosis may be considered for extensive therapeutic intervention to suppress inflammation across multiple organs.

The levels of NLR and PLR in patients with psoriasis have been found to be elevated across different racial groups. A study of 186 patients with PsV and 50 patients with PsA revealed that NLR and PLR levels decreased in parallel with CRP in Japanese psoriasis patients, regardless of the type of biologic therapy used [18]. Another study conducted on 111 patients with PsV and 25 patients with PsA in Korea showed that NLR, PLR, and ESR were statistically significant predictors of PsA, with NLR being the strongest predictor (odds ratio = 3.351, *p* = 0.005) [34]. In a retrospective analysis of psoriasis patients in China, Egypt, and Turkey, NLR and PLR were also found to be elevated and correlated with disease severity [35,36,37]. The combination of NLR and PLR can predict adverse events in patients with acute myocardial infarction and prognosis of malignant tumors [38,39]. There is no consistent trend as to which marker, NLR or PLR, has a stronger association with disease severity or predicts systemic inflammation more accurately. Thus, this combination may also be beneficial in predicting disease severity or treatment response in psoriasis. Given that SII, a multiplier of neutrophil and PLR, incorporates elements of both neutrophils and platelets, this marker may become a more promising prognostic factor. SII was found to be a useful predictor of treatment persistence for conventional systemic agents in the present study; however, it was not clear whether SII was a better predictor than other factors such as neutrophil or platelet counts, or PLR. The significance of SII needs to be further explored in a cross-racial, multicenter study with a larger sample size.

The present study has suggested platelets and neutrophils as potential contributors to psoriatic systemic inflammation. While platelets are primarily recognized for their role in hemostasis, recent evidence has increasingly highlighted their role in the regulation of inflammation and immunity [40,41]. Elevated platelet counts in circulation may result from increased bone marrow hematopoiesis as a compensatory response to platelet accumulation at inflammatory sites. In addition, cytokines such as TNF, which are increased at inflammatory sites, directly activate platelets, further promoting the development of thrombosis and cardiovascular diseases with enhanced inflammation [42,43,44]. Regarding neutrophils, their abundance in the epidermal stratum corneum is a typical histopathological feature of psoriasis [45]. Neutrophils migrate to psoriatic lesions and enhance inflammation by promoting the production of oxidative stress and the formation of neutrophil extracellular traps, which are associated with both the development and maintenance of psoriasis [46,47,48]. This study has demonstrated the clinical relevance of platelets and neutrophils, suggesting their importance in inflammation and immune regulation in psoriasis.

Biologics are potent drugs with long-term efficacy and are powerful agents that can reduce systemic inflammation [49,50]. In fact, it has been reported that these inflammatory scores of NLR and PLR decrease after treatment with biologics in psoriasis [18], and thus, systemic treatment with biologics are suitable drugs for reducing systemic inflammation based on the concept of PsD. The present study found that patients who initiated biologics exhibited higher overall drug persistence, regardless of pretreatment blood data. However, some patients treated with infliximab showed a tendency to switch drugs, which could be partially due to the potential immunogenicity of the drug. A larger sample size may be needed to determine whether there is a difference in anti-inflammatory efficacy among biologics.

There are several limitations in the present study: the sample size was small, and the analysis was performed at a single center. Second, the analysis was limited to a short follow-up period of one year to examine the drug continuation rate. Since the systemic treatments of biologics have the advantages of long-term efficacy, a longer follow-up study would be desirable. In addition, elucidating the pathogenic mechanism by which elevated platelet or neutrophil counts, PLR, and SII levels indicate poor treatment response is beyond the scope of this study, and further findings, including animal studies, are expected.

In conclusion, PLR and CRP are associated with the diagnosis of PsA, and patients with higher platelet or neutrophil counts or PLR and SII scores are more resistant to treatment with conventional systemic agents. Regular monitoring of inflammatory score trends is recommended for these patients. Prospective randomized studies to determine the change in systemic inflammation scores and the improvement in comorbidities with each systemic drug will allow us to evaluate which markers are promising in reducing systemic inflammation depending on patients’ comorbidities. This will lead us to further understand a comprehensive concept of PsD and enable personalized medicine in the future.

## Figures and Tables

**Table 1:** Baseline characteristics and inflammatory biomarkers.

	PsV (N = 117)	PsA (N = 47)	Healthy (N = 50)	*p* Value		
				PsV vs. Healthy	PsA vs. Healthy	PsV vs. PsA
Age, years	51.16 ± 18.26	55.49 ± 13.78	54.30 ± 13.62	0.6302	>0.9999	0.4962
Male, %	68	62	64	0.5938	0.8364	0.4658
PASI scores	10.66 ± 10.63	9.18 ± 8.48				0.6310
Neu (×10^9^/L)	4.294 ± 1.345	4.787 ± 2.123	4.154 ± 2.018	0.2908	0.0641	0.6745
Lym (×10^9^/L)	1.865 ± 0.665	1.780 ± 0.712	1.744 ± 0.590	0.4901	>0.9999	0.8067
Mono (×10^9^/L)	0.407 ± 0.128	0.437 ± 0.154	0.368 ± 0.111	0.1182	0.0179	0.5777
Platelet (×10^9^/L)	259.52 ± 71.14	289.85 ± 90.13	272.06 ± 63.668	0.5466	0.4657	0.0324
NLR	2.704 ± 1.659	3.415 ± 3.477	2.614 ± 1.53	>0.9999	0.4631	0.3745
MLR	0.247 ± 0.134	0.294 ± 0.220	0.227 ± 0.082	>0.9999	0.3714	0.4092
PLR	154.9 ± 65.11	196.3 ± 125.29	172.116 ± 66.244	0.3601	>0.9999	0.2232
SII	687.1 ± 445.2	1105 ± 1515	733.020 ± 594.958	>0.9999	0.4281	0.1560
SIRI	1.13 ± 0.85	1.74 ± 3.06	1.019 ± 0.964	0.7774	0.0711	0.2715
CRP (mg/dL)	0.29 ± 0.57	1.48 ± 4.84				0.0284

Significance was determined by Fisher’s exact test for sex distribution, by Mann–Whitney’s U-test for PASI scores, and by Kruskal–Wallis test with Dunn–Bonferroni post hoc test for other items. Values are shown by mean ± SD. NLR, neutrophil-to-lymphocyte ratio; MLR, monocyte-to-lymphocyte ratio; PLR, platelet-to-lymphocyte ratio; SII, systemic immuno-inflammatory index; SIRI, systemic inflammatory response index; CRP, C-reactive protein.

**Table 2:** Cut-off values and AUC from ROC curves for discriminating PsV and PsA.

	Cut-Off Values	AUC	Sensitivity	Specificity
NLR	3.211	0.57012	0.3913	0.7683
MLR	0.208	0.56747	0.6739	0.4878
PLR	275	0.57887	0.2826	0.939
SII	911.6	0.59213	0.4348	0.7805
SIRI	0.870	0.57542	0.6522	0.5122
CRP (mg/dL)	0.30	0.58643	0.4130	0.7927

Cut-off values were determined by the Youden Index calculated from the ROC curves. NLR, neutrophil-to-lymphocyte ratio; MLR, monocyte-to-lymphocyte ratio; PLR, platelet-to-lymphocyte ratio; SII, systemic immuno-inflammatory index; SIRI, systemic inflammatory response index; CRP, C-reactive protein.

**Table 3:** Univariate and multivariate Cox proportional hazards model analysis predicting disease diagnosis of PsA by systemic inflammatory markers.

Factors		Univariate		Multivariate	
		OR (95 % CI)	*p* Value	OR (95 % CI)	*p* Value
NLR	≥3.211	2.184 (0.918–5.194)	0.077	0.579 (0.134–2.498)	0.464
MLR	≥0.208	2.355 (1.044–5.316)	0.039	2.789 (0.845–9.201)	0.092
PLR	≥275	5.775 (1.698–19.643)	0.005	7.027 (1.089–45.348)	0.040
SII	≥911.6	1.682 (0.759–3.728)	0.200	0.506 (0.139–1.843)	0.302
SIRI	≥0.870	2.423 (1.024–5.729)	0.044	0.915 (0.227–3.682)	0.901
CRP (mg/dL)	≥0.30	3.251 (1.379–7.716)	0.008	3.179 (1.183–8.538)	0.022

OR, odds ratio; CI, confidence interval; NLR, neutrophil-to-lymphocyte ratio; MLR, monocyte-to-lymphocyte ratio; PLR, platelet-to-lymphocyte ratio; SII, systemic immuno-inflammatory index; SIRI, systemic inflammatory response index; CRP, C-reactive protein.

**Table 4:** Characteristics and systemic inflammatory markers among patients with psoriatic arthritis with or without axial lesions.

	With Axial Lesions	Without Axial Lesions	*p* Value
Age, years	55.5 ± 14.8	55.5 ± 12.9	0.8699
Male, %	64	59	0.7712
PASI scores	12.02 ± 9.75	6.05 ± 5.52	0.0227
Neutrophil count (×10^9^/L)	4.96 ± 2.59	4.60 ± 1.50	0.7894
Lymphocyte count (×10^9^/L)	1.88 ± 0.84	1.67 ± 0.54	0.5518
Monocyte count (×10^9^/L)	0.44 ± 0.17	0.43 ± 0.14	0.8643
Platelet count (×10^9^/L)	295.72 ± 100.06	283.18 ± 79.14	0.5019
NLR	3.68 ± 4.53	3.13 ± 1.80	0.4887
MLR	0.31 ± 0.28	0.28 ± 0.12	0.5820
PLR	199.13 ± 148.26	193.20 ± 97.69	0.6869
SII	1288.77 ± 2031.87	903.87 ± 562.58	0.7522
SIRI	2.08 ± 4.15	1.36 ± 0.96	0.6200
ESR (mm/h)	28.28 ± 36.00	16.10 ± 18.02	0.8397
CRP (mg/dL)	2.60 ± 6.55	0.26 ± 0.53	0.0271

Significance was determined by Fisher’s exact test for sex distribution and by Mann–Whitney’s U-test for other items. Values are shown by mean ± SD. NLR, neutrophil-to-lymphocyte ratio; MLR, monocyte-to-lymphocyte ratio; PLR, platelet-to-lymphocyte ratio; SII, systemic immuno-inflammatory index; SIRI, systemic inflammatory response index; ESR, erythrocyte sedimentation rate; CRP, C-reactive protein.

**Table 5:** The number of patients initiating each systemic therapy and concomitant treatments during the one-year follow-up.

Treatment		Number of Patients	Concomitant Treatments during the One-Year Follow-Up
Biologics (N = 51)	Infliximab	9	None
	Adalimumab	2	None
	Certolizumab Pegol	0	None
	Secukinumab	11	One patient received cyclosporine. One patient received apremilast.
	Ixekizumab	5	None
	Brodalumab	1	None
	Bimekizumab	0	None
	Guselkumab	8	None
	Risankizumab	11	None
	Tildrakizumab	4	None
	Ustekinumab	0	None
Conventional systemic agents (N = 48)	Methotrexate	8	One patient received cyclosporine.
	Etretinate	6	One patient received apremilast.
	Cyclosporine	3	None
	Apremilast	31	One patient received etretinate. One patient received secukinumab.

**Table 6:** Kaplan–Meier analyses for treatment continuation rates of patients who received treatment with biologics and by log-rank tests in patients with high and non-high scores for peripheral blood parameters and systemic inflammatory markers.

	Cut-Off Values	Median Treatment Duration (Days)		Log-Rank Test
		High-Score Group	Non-High-Score Group	*p* Value
Neu (×10^9^/L)	8.19	365	365 ± 102.48	0.6483
Lym (×10^9^/L)	2.92	308.5 ± 81.79	365 ± 104.89	0.8662
Mono (×10^9^/L)	0.59	365 ± 27.98	365 ± 108.76	0.6100
Platelet (×10^9^/L)	399.40	365	365 ± 102.48	0.6483
NLR	5.674	365	365 ± 104.54	0.3418
MLR	0.391	365 ± 68.99	365 ± 106.45	0.5994
PLR	304.6	365	365 ± 102.48	0.6483
SII	1923	365	365 ± 103.16	0.5133
SIRI	2.95	365 ± 20.78	365 ± 104.18	0.5638
CRP (mg/dL)	0.69	365 ± 98.84	365 ± 102.32	0.3290

Patients were divided into two groups according to cut-off values. Cut-off values were determined by mean + 2SD levels of healthy controls. NLR, neutrophil-to-lymphocyte ratio; MLR, monocyte-to-lymphocyte ratio; PLR, platelet-to-lymphocyte ratio; SII, systemic immuno-inflammatory index; SIRI, systemic inflammatory response index; CRP, C-reactive protein.

**Table 7:** Kaplan–Meier analyses for treatment continuation rates of patients who received treatment with oral apremilast, methotrexate, cyclosporine, and etretinate by log-rank tests in patients with high and non-high values for peripheral blood parameters and systemic inflammatory markers.

	Cut-Off Values	Median Treatment Duration (Days)		Log-Rank Test
		High-Score Group	Non-High-Score Group	*p* Value
Neu (×10^9^/L)	8.19	23 ± 25.46	147 ± 142.22	<0.0001
Lym (×10^9^/L)	2.92	166	120.5 ± 145.10	0.5101
Mono (×10^9^/L)	0.59	63 ± 167.27	147 ± 139.52	0.3175
Platelet (×10^9^/L)	399.40	50 ± 127.98	166 ± 142.58	0.0006
NLR	5.674	41 ± 198.27	134.5 ± 141.10	0.1335
MLR	0.391	59 ± 144.21	156.5 ± 143.01	0.1053
PLR	304.6	59 ± 111.23	156.5 ± 145.12	0.0107
SII	1923	59 ± 162.92	134.5 ± 142.47	0.0383
SIRI	2.95	41 ± 198.27	134.5 ± 141.10	0.1335
CRP (mg/dL)	0.69	280 ± 156.94	122 ± 144.04	0.1303

Patients were divided into two groups according to cut-off values. Cut-off values were determined by mean + 2SD levels of healthy controls. NLR, neutrophil-to-lymphocyte ratio; MLR, monocyte-to-lymphocyte ratio; PLR, platelet-to-lymphocyte ratio; SII, systemic immuno-inflammatory index; SIRI, systemic inflammatory response index; CRP, C-reactive protein.
